# Visceral leishmaniasis: Clinical and demographic features in an African population

**DOI:** 10.12669/pjms.292.3151

**Published:** 2013-04

**Authors:** Abdelsalam M. Nail, Abdelmageed M. Imam

**Affiliations:** 1 Abdelsalam M. Nail, MBBS, MD, MSc (Tropical Medicine), Department of Medicine, College of Medicine,; 2 Jouf University, Jouf, Kingdom of Saudi Arabia. Abdelmageed M. Imam, MBBS, PhD, DTM&H, Parasitology Unit, College of Medicine, Qassim University, Qassim, Kingdom of Saudi Arabia.

**Keywords:** Visceral leishmaniasis, African population, Clinical features

## Abstract

***Objective:*** To describe the clinical and demographic features of patients with visceral leishmaniasis (VL), from Sudan, Africa.

***Methodology:*** A descriptive study was conducted during 5 years period on confirmed VL patients. These patients are, originally, from White Nile Province (WNP) region, a previously non-endemic VL focus which is located in southern part of Sudan. They were referred during the period 2006-2010 for management at Tropical Diseases Hospital (TDH) in the capital Khartoum. The patients data were retrieved from the hospital electronic software system, and were studied.

***Results:*** A total of 71 patients with VL were reviewed. The main clinical features were: fever 68 (95.8%), splenomegaly 66 (93%), weight loss 61 (85.9%), pallor 59 (83.1%), hepatomegaly 52 (73.2%). The most notable haematology finding was the mean Hb value (7.6 g/dL) on admission to hospital. Gender showed males at higher risk for VL as compared to females with a ratio of 3:1 (53 vs 18). VL was largely a disease of children with 42 (59.1%) aged < 15 year, and around quarter (23.9%) under 5 years.

***Conclusion:*** The clinical features of Sudanese VL in WNP region is, generally, similar to the pattern seen globally in endemic foci. The majority of the study population are paediatric indigenous VL patients, suggesting that adults were immune, and indicating change of disease pattern from previous sporadic to present endemic. This finding emphasizes the need for research to better understand VL in non-endemic areas with the objective of developing effective and sustainable control strategies.

## INTRODUCTION

*Visceral leishmaniasis (VL), also known as kala-azar, is a protozoal parasitic disease caused by *Leishmania donovani *complex, and transmitted to man by the bite of infected female sandfly *Phlebotomus* spp. in Africa.*^1^* VL is an infection of the reticulo-endothelial system characterized clinically by a chronic febrile course, and associated with loss of weight and hepato-splenomegaly. The diagnosis is usually established by identification of the parasite in aspirate material from bone marrow, spleen, or lymph nodes. The World Health Organization estimates the annual global rate for VL prevalence and incidence at 2.5 and 0.5 million cases, respectively. VL causes 60-70 thousand deaths every year.*^2^

*Many factors have contributed to the worldwide increased interest in basic and clinical research on VL. First, VL is a vector-borne disease which is adapting to environment changes, and spreading into urban and suburban areas, such as in Brazil.* ^3^ *Second, VL is recognized as a major opportunistic infection associated with HIV/AIDS patients, such as in Ethiopia.* ^4^ *Third, the expense and side effects of drug therapy, the lack of an effective vaccine, the diversity of the sand fly biology, and the changing virulence behaviour of the causative protozoan: continue to represent a challenge in the understanding and management of VL. Finally, global warming is being suggested as a possible reason for the eventual spread of diseases now seen primarily in the tropics, such as VL, to more temperate climates.* ^5^ *An interesting report from Britain is presuming that VL may become endemic in southern England, based on a prediction that by the year 2025 that area will have a climate like that currently seen in the south of France where VL disease and vector are present.* ^6^

*VL is an important health problem in east African countries including Kenya, South Sudan, Uganda, Ethiopia, Somalia, and Sudan.* ^7^^-^^10^ *In Kenya, VL is encountered mainly in the region of Baringo, however, changing improved lifestyle has led to a decreased incidence rate of VL among males in this country. In West Pokot region of Uganda, the majority of VL patients belong to the paediatric age group. In Ethiopia, VL is encountered mostly as an opportunistic infection in HIV/AIDS adult patients, and the mortality rate is relatively high. In South Sudan, most of VL cases were reported from Upper Nile Province region during a large epidemic outbreak that started in the year 1984 and claimed around 100 thousands of human lives. In Somalia, VL has been reported from the region of Shebelle River in the south of the country. Transmission of VL to man in this country is believed to be mainly anthroponotic (from a human reservoir).*

*In Sudan, the first VL patient was described in the year 1904. Transmission dynamics are believed to include both zoonosis (from an animal reservoir) as well as anthroponosis. The sandfly vector is *Phlebotomus orientalis*. The leishmania* *isolated from human and sandfly possess three zymodemes: MON-18, MON-30, and MON-82 which all belong to *Leishmania donovani *complex.*^1^ *The typical clinical features include: fever, weight loss, hepato-splenomegaly, and pancytopenia. Post kala-azar dermal leishmaniasis (PKDL), a skin complication of VL encountered after or during treatment, has been reported in up to 58% of patients.*^11^ *Genetic susceptibility is an epidemiologic feature in Sudanese VL.*^12^ *This genetic feature was suggested following observations of marked variations in incidence of clinical disease among populations of different ethnicities sharing the same immediate environment and transmission exposures.*

*All patients of the present study are, originally, from the White Nile Province region (WNP) in southern part of Sudan. The WNP region is 120km south of the capital Khartoum where the patients were referred to, and managed in Tropical Diseases Hospital (TDH). Studies on VL from the WNP region are scarce, and in this report we present our findings on the clinical and demographic features of VL patients from this region.*

## METHODOLOGY

This is a descriptive cross sectional hospital-based study. The study population are 71 VL patients who are originally from rural areas in WNP region which is located in southern part of Sudan. The patients were managed in TDH in the capital Khartoum. TDH is a Ministry of Health referral hospital, and affiliated to Faculty of Medicine, Omdurman Islamic University, Sudan. This hospital was founded in 1974 for research, service, and training on tropical diseases, mainly visceral leishmaniasis, malaria, and schistosomiasis. The Quality Control Unit of TDH is operated by trained medical officers who verify the documentation and transfer of patient data from the hospital medical records into an electronic software system.

Confirmation of diagnosis for VL in the study patients was carried out by (1) Demonstration of amastigote stage of parasite in aspirate material from lymph node, bone marrow, or spleen using direct light microscopy or (2) Positive serology using Direct Agglutination Test (DAT) at a significant titer of 1:6400 or more, combined with a negative Leishmanin Skin Test (LST). The clinical features and demography of confirmed VL patients during the period 2006-2010, were retrieved from the hospital records electronic system. Next, the patients’ information was entered onto a standardized data sheet developed by us. The data included VL symptoms, signs, laboratory findings, drug therapy, patient gender, and age.

Computer assisted analysis, using statistical program for social sciences (SPSS) version 16, was employed. The study was approved by the Research and Ethics Committee of Faculty of Medicine, Omdurman Islamic University, Sudan.

## RESULTS

Table-I shows the clinical symptoms of the 71 VL study patients. Fever was encountered in 68 (95.8%) of cases. The duration of fever was calculated in weeks, and ranged between 1-72 weeks with a mean of 10.8 ± 11.3 standard deviation (SD). Other symptoms encountered were abdominal pain (67.6%), loss of appetite (56.3%), weight loss (85.9%), epistaxis (29.6%), diarrhea (25.4%), cough (39.4%) and joint pains (29.6%).

Table-II shows the clinical signs of the 71 VL study patients. Pallor (83.1%), skin pigmentation (8.5%), lymphadenopathy (50.7%), splenomegaly (93%), hepatomegaly (73.2%), ascites (9%), and lower limbs oedema (14.1%). In the patients who had splenomegaly; the median spleen size on admission to hospital was 8 cm (range, 2cm – 26cm).

Table-III shows the gender and age distribution of the 71 VL study patients. Males are at higher risk for VL as compared to females with a ratio of 3:1 (53 vs 18). VL was largely a disease of children with 42 (59.1%) occurring among those aged < 15 years. Around quarter of the patients (23.9%) were under 5 years of age.

Table-IV shows the haematology findings of the 71 VL study patients. The most notable feature was anaemia which was found in 77% of patients. They had Hb < 11g/dL. The mean value of haemoglobin among all the study patients was 7.6 g/dL on admission to hospital.

The results of drug outcome showed that all patients received sodium stibogluconate (SSG) with a cure response in 67 (94.4%) cases. Four patients died giving a case fatality rate of 5.6%.

**Table-I:** Clinical Symptoms in 71 African visceral leishmaniasis patients

*Symptom*	*Number*	*%*
Fever	68	95.8
Weight loss	61	85.9
Abdominal pain	48	67.6
Loss of appetite	40	56.3
Cough	28	39.4
Epistaxis	21	29.6
Joint pain	21	29.6
Diarrhoea	18	25.4

**Table-II:** Clinical Signs in 71 African visceral leishmaniasis patients

*Sign*	*Number*	*%*
Splenomegaly	66	93.0
Pallor	59	83.1
Emaciation	54	76.1
Hepatomegaly	52	73.2
Lymphadenopathy	36	50.7
Oedema of lower limbs	10	14.1
Skin darkness	06	08.5
Ascites	06	08.5

**Table-III:** Gender and Age of 71 African visceral leishmaniasis patients

*Gender *	*Number*	*%*
Male	53	74.6
Female	18	25.4
*Age*		
1- 5	17	23.9
5-10	12	16.9
10-15	13	18.3
15-20	09	12.7
20-25	09	12.7
25-30	08	11.3
30-35	02	02.8
35-40	01	01.4

**Table-IV:** Haematology Findings in 71 African visceral leishmaniasis patients

*Hb g/dL*	*Value*	*%*
1- < 5.7	16	22.2
2- 5.7 - 11.4	50	70.8
3- 11.4 - 14.2	01	01.4
4- > 14.2	04	05.6
*WBCs/mm* ^3^		
1-< 2000	21	29.2
2- 2001 – 4000	26	36.2
3- > 4001	22	34.6
*Platelets/mm* ^3^		
1-<40000	03	04.2
2- 40001 – 60000	09	12.5
3- 60001 – 80000	10	13.9
4- 80001 – 100000	16	26.2
5- 100001 – 120000	12	16.7
6- >120001	18	26.5

## DISCUSSION

*Before the year 1988, VL did not exist in WNP region, southern Sudan. The Upper Nile Province region which is bordering WNP region southward had experienced a large epidemic outbreak of VL that started during 1984 and went on until 1994.* ^1^ *Retrospect analysis of population migration has supported the assumption that VL of WNP region was a spread from their neighbouring region. Further support was provided by the concept that VL is an emerging disease where the sandfly vector and the protozoal parasite are able to adapt and spread into intra-domiciliary and peri-urban environments.* ^13^ *In Brazil, it was documented that during the late 1980s VL has expanded into previously unaffected rural areas and into the outskirts of large cities.*^14^ *This situation has resulted into undue delay in diagnosis of VL, as well as rise in mortality rates in urban VL cases, because the disease was under-estimated by medical practitioners. In Nepal, VL case reports were found to be rising in areas of the country previously not recognized as endemic.*^15^

*The WNP region is located in southern part of Sudan, around 120 km south of the capital Khartoum, and bordering northward of Upper Nile Province region, a known VL endemic area in Sudan. The climate in WNP region is essentially subtropical, and agriculture is an important part of the population activities, with abundant *Acacia seyal* and *Balanites aegyptica* vegetations.*^16^* Together with other socio-demographic factors, these climate characteristics favour VL transmission among local populations. The first documented cases of VL from WNP region (16 in total) were described in 1988 among local inhabitants of small villages.*^17^* Most of the subsequent cases showed a paediatric age profile, suggesting that adults are immune, and indicating an endemic pattern of VL. Thus, in retrospect analysis, we understand that VL cases in WNP region were sporadic case reports from a non-endemic area, during the late 1980s through into the 1990s, following the large epidemic outbreak in the neighbouring region. Next, the appearance of clusters of VL among children during the second half of the decade starting the year 2000 (covering the present study period) has provided evidence that the disease has changed to the pattern of an endemic area.*

*The clinical features of VL patients, in this study, are more or less similar to the disease pattern in South America and Asia.* ^18^^,^^19^ *Fever, hepato-splenomegaly, loss of weight, and pancytopenia are the classic features of VL which were manifest in the majority of our patients, at the time of hospital admission,* Table I *,* II *, and* IV *. VL in WNP region is, relatively, of recent encounter to medical practitioners in this region, and they used to under-estimate VL and diagnose it as malaria, which is a febrile illness associated with splenomegaly, and endemic in the region. This situation explains the undue delay in VL diagnosis and the late presentation of patients (data not shown) to TDH referral hospital in Khartoum. Comparable to our finding, a study from Pernambuco region in north-eastern Brazil has suggested childhood febrile illnesses are being considered as initial diagnoses in urban VL patients.* ^20^ *Consequently, significant delay of VL diagnosis was encountered and had resulted in increase of mortality rates in that region.*

*Lymphadenopathy is a common VL clinical feature, described not only in this study, but also in previous literature from east Africa.* ^21^ *Although this feature is classically labelled as rare in Indian VL, a recent report from Himalayan region (previously non-endemic area), described lymphadenopathy in a series of 18 patients.*^22^* These inter-continental variations are, partly, explainable in terms of genetic factors. Evidence is accumulating that genetic diversity is playing a role in susceptibility to incidence of VL disease and to the severity of its clinical features. For example, in Sudan, tribes of different ethnicities who shared the same immediate environment and same VL transmission exposures were found to show marked variations as regard incidence of clinical disease. A prospective study on these different tribes, using multi-case families, has demonstrated an association link between the gene *SLC11A1* (formerly *NRAMP1*) that codes for innate resistance, and incidence of clinical VL.*^12^* An interesting recent case-control study from India, has demonstrated a single point gene association between clinical VL, and *FAM120B *gene on chromosome 6q27.*^23^* These findings confirm the potential for genetic polymorphism to act as risk factor for acquisition of VL. Similarly, PKDL, has been described to show an incidence rate as low as 5-10% in Indian VL patients, while an incidence rate as high as 58% was reported in Sudanese VL patients.*^24^ *Polymorphism at *IFNGR1 *gene* *was found to be associated specifically with PKDL disease susceptibility among Sudanese populations.*^12^

*Male patients, in this study, were at higher risk for VL as compared to females with a ratio of 3:1 (53 vs 18). This male gender preponderance, particularly among children, has also been reported from South America.* ^25^ *However; the underlying aetiology of this VL feature is not fully understood. A study from Brazil has suggested a hormonal factor to be linked with this phenomenon.* ^26^ *We suggest a potential theoretical risk factor exposing Sudanese male children to sandfly bites: the local social tradition of male children in rural areas who go outdoors for play activities, while females prefer indoor playing.*

*While the present study contributes important information from Africa to the concept that VL is a disease able to spread and adapt to changing environments, there are some important limitations. First, being a hospital-based study, the actual magnitude of VL cases could have been under-reported. Second, molecular characterization of leishmania isolates, from humans, animal reservoir, and sandfly vector in WNP region and its neighbouring endemic region is important to add to the clinical and epidemiologic evidence of disease interactions.*

## CONCLUSION

The present study shows that the clinical features and demography pattern of Sudanese VL are, generally, similar to the picture seen in endemic areas worldwide. Also we described a situation of a VL area with previous sporadic case reports changing into endemic disease pattern. This finding emphasizes the need for research to better understand VL behaviour in non-endemic areas with the objective of developing effective and sustainable control strategies.
